# The HIKCUPS trial: a multi-site randomized controlled trial of a combined physical activity skill-development and dietary modification program in overweight and obese children

**DOI:** 10.1186/1471-2458-7-15

**Published:** 2007-01-31

**Authors:** Rachel A Jones, Anthony D Okely, Clare E Collins, Philip J Morgan, Julie R Steele, Janet M Warren, Louise A Baur, Dylan P Cliff, Tracy Burrows, Jane Cleary

**Affiliations:** 1 Child Obesity Research Centre, University of Wollongong, Wollongong, NSW, 2522, Australia; 2 Faculty of Education, University of Wollongong, Wollongong, NSW, 2522, Australia; 3 Faculty of Health, University of Newcastle, Newcastle, NSW, 2308, Australia; 4 Faculty of Education, University of Newcastle, Newcastle, NSW, 2308, Australia; 5 Biomechanics Research Laboratory, University of Wollongong, Wollongong, NSW, 2522, Australia; 6 University of Sydney Discipline of Paediatrics & Child Health Sydney The Children's Hospital at Westmead, Sydney, 2145, Australia; 7 Children's Nutrition Research Centre, Royal Children's Hospital, Herston, Queensland, 4029, Australia; 8 Department of Clinical Nutrition, Wollongong Hospital, Wollongong, NSW, 2500, Australia

## Abstract

**Background:**

Childhood obesity is one of the most pressing health issues of our time. Key health organizations have recommended research be conducted on the effectiveness of well-designed interventions to combat childhood obesity that can be translated into a variety of settings. This paper describes the design and methods used in the Hunter Illawarra Kids Challenge Using Parent Support (HIKCUPS) trial, an ongoing multi-site randomized controlled trial, in overweight/obese children comparing the efficacy of three interventions: 1) a parent-centered dietary modification program; 2) a child-centered physical activity skill-development program; and 3) a program combining both 1 and 2 above.

**Methods/Design:**

Each intervention consists of three components: i) 10-weekly face-to-face group sessions; ii) a weekly homework component, completed between each face-to-face session and iii) three telephone calls at monthly intervals following completion of the 10-week program. Details of the programs' methodological aspects of recruitment, randomization and statistical analyses are described here *a priori*.

**Discussion:**

Importantly this paper describes how HIKCUPS addresses some of the short falls in the current literature pertaining to the efficacy of child obesity interventions.

The HIKCUPS trial is funded by the National Medical Research Council, Australia.

## Background

The prevalence of childhood obesity is increasing in many countries throughout the world and is associated with immediate and long-term medical, psychological and psychosocial complications [1] making it one of the most pressing public health problems of our time [2]. Key health and medical organizations in several countries have recommended research be conducted on the effectiveness of well-designed interventions to combat childhood obesity that can be translated into a variety of settings [3-6].

To date, conventional weight management strategies for childhood obesity have only shown at best moderate success short-term [7]. Few studies have looked at intervention effects in the medium or long-term [8]. Furthermore, most of the randomized controlled trials (RCTs) identified in recent systematic reviews of child and adolescent obesity treatment and prevention programs have been performed in highly resourced tertiary management settings and, therefore, lack evidence that they would be transferable to community settings. In general, these previous RCTs have also had inadequate follow-up periods; small sample sizes with sometimes broad ages ranges of children; high drop-out rates; lack of intention-to-treat analyses; and did not assess important secondary outcomes such as changes in clinical risk factors [7,8].

In order to address some of the shortfalls in the current literature related to managing pediatric obesity, a on-going RCT, the Hunter Illawarra Kids Challenge Using Parents Support (HIKCUPS) trial, was designed to treat childhood overweight and obesity.

## Study aim

The aim of the HIKCUPS trial is to compare and evaluate the efficacy of the following three intervention strategies in overweight and obese 5–9-year-old children:

1. a parent-centered dietary modification program;

2. a child-centered physical activity skill development program; and

3. a combination of both the parent-centred dietary modification and the child-centred physical activity skill-development program.

## Methods and design

### Ethical considerations

The Universities of Wollongong and Newcastle Human Ethics Research Committee have approved this study. Written informed consent from all parents was sort prior to their enrolment into the study.

### Study interventions

#### 1. The parent-centered Dietary Modification program (DM)

The DM program was designed specifically for parents, as parents of young children play a pivotal role in facilitating changes in a child's food choice, intake and behaviors and are the major role models for healthy lifestyle behaviors [9]. The DM program is based on the Health Belief Model and assumes that parents will make the recommended actions to modify the food behaviors relevant to their family members if they feel that doing so will help to control their child's weight problems and avoid obesity-associated complications [10]. It emphasizes making small changes daily, building on success and developing supporting factors to achieve a sustainable healthy family-eating pattern. Goal setting, problem-solving, role-modeling and positive reinforcement are used to manage changes in food behaviours and strategies incorporated to help parents increase their confidence in making changes related to their goals. The structure and content of the program uses a cognitive behavioral, solution-focused approach from the emerging field of health coaching [11-13].

The DM program has three major components:

##### (i) Parent focused face-to-face group sessions

This consists of ten 2-hour face-to-face weekly sessions. Behavioural change is targeted during the sessions to reduce total energy and fat intakes, increase fruit and vegetable intake and make healthy beverage and snack choices. Practical advice about food shopping and preparation is provided with the sessions including a diadactic component, group work and practical activities.

##### (ii) Homework

During the 10-weeks parents are encouraged to implement SMART (specific, measurable, achievable, realistic and time-framed) goals into their home environment [14,15]. These goals, although small, result in important practical changes. Facilitators also encourage all group members to share their weekly successes and challenges with other parents in the program thereby facilitating group empowerment and motivation.

##### (iii) Follow-up

In the final week of the face-to-face sessions, members are asked to document several short- to medium-term SMART goals that they can work on. They are also encouraged to identify barriers in the lives of themselves and their children that might prevent them from achieving these goals. Group members are asked to record their goals on both a goal setting chart, which they take home at the end of the session and a postcard, which is mailed to them six months later. The facilitators telephone parents monthly for the first three months after the face-to-face group sessions to discuss their progress in meeting these goals. Each telephone session is structured using a REGROW (Review, Evaluate, Goal, Reality, Options, Wrap-up) model of coaching [15]. These telephone sessions aim to review and evaluate existing goals, explore what is happening in reality, discuss possible options and wrap-up the session by documenting new goals.

#### 2. The child-centered Physical Activity Skill-Development program (PASD)

The physical activity skill-development program (PASD) is based on Competence Motivation Theory [16] modified for the physical domain [17]. Competence Motivation Theory posits that children's motivation to participate in physical activity is influenced by their actual and perceived physical competence, social support, and enjoyment of physical activity [16]. The PASD program focuses on increasing the children's actual competence in performing fundamental movement skills, perceived competence, and encouraging and improving the level of social support provided to children in their physical activity endeavours.

Like the DM program, the PASD program has three components:

##### (i) Child-focused group sessions

Children attend ten 2-hour face-to-face weekly sessions. Each week children participate in a variety of activities aimed at improving their mastery of 12 fundamental movement skills (run, jump, leap, hop, slide, gallop, strike, roll, kick, throw, catch, bounce). Each session covers three fundamental movement skills, such that over the course of the 10-weeks each skill is re-visited, although the focus is on more complex components of the skill, in subsequent sessions. Skill mastery is aided by adherence to lesson plans for each skill incorporating several learning stages:

a) contextual stage (questions children as to what games, sports and activities require mastery of the specific skill and how the skill is performed proficiently);

b) exploration stage (allows children to explore the different movement patterns related to the skill using movement concepts such as force, speed, levels and relationships);

c) guided discovery stage (isolates specific components of a skill and using a problem solving approach, guides children to discover the correct way to perform the skill); and

d) skill application stage (applies the skills in small drill activities and modified game contexts).

The various activities included in the program have been purposefully selected and adapted to enable children to experience success in their skill practice in a non-threatening and supportive learning environment. Facilitators utilise key pedagogical strategies to ensure students have fun, improve their skill performance and are motivated to practice. The activities conducted in the PASD program have accessed from a variety of resources [18,19] and are also based on the experience of several of the authors and facilitators who have a number of years of primary school teaching experience.

##### (ii) Homework

In order to maximise the children's competence and confidence, they are strongly encouraged to practice the fundamental movement skills at home with their parents and/or siblings, between each group session. Each participant is given a 'Home-challenge folder', which includes fun, relevant and developmentally appropriate activities enabling practice of skills at home. The home challenges take approximately 30 mins and children are encouraged to complete three sessions each week. The importance of the home challenges and the parent/sibling involvement in the program is explained to the parents during a 1 hour workshop held by the facilitators in the first group session. A sticker chart and certificates are used as incentives to maximise adherence to the home challenges.

##### (iii) Follow-up

In the final session of the program, parents attend another 1 hour workshop where they are encouraged to set realistic short- to medium-term SMART goals for increasing physical activity and reducing sedentary behaviours. They are asked to identify barriers in their family lives that may prevent their child from participating in sufficient physical activity or that leads to their child spending excessive amounts of time in small screen recreation (e.g. watching television, videos or DVDs, playing computer games or using the computer for fun). As described in the DM program, parents then write down several short- to medium-term SMART goals on goal setting charts and postcards. Families are telephoned monthly for three months to discuss and re-evaluate these goals with discussion based on the REGROW model of coaching. Additionally children attend a 2 hour 'refresher' session two months after the final session where all the fundamental movement skills are revised. In this session the key components of each skill are again reinforced through modified and minor games and the importance of practising each skill is reiterated, along with the importance of staying active and having fun whilst being active.

#### 3) The DM/PASD program

The third intervention involves a combination of the DM and PASD programs whereby the children participate in the PASD program at the same time as their parent(s) participate in the DM program.

### Outcome measures

The primary outcome measures for the HIKCUPS trial are Body Mass Index (BMI) *Z*-score and waist circumference. The secondary outcomes include: metabolic profile (blood pressure and fasting levels of total cholesterol, HDL and LDL cholesterol, triglycerides, glucose, insulin and C-reactive protein), dietary intake (mean daily intake or core and non-core foods) and Child Feeding Questionnaire [20], fundamental movement skill proficiency and perceived competence, objectively measured physical activity, time spent in sedentary activities, proficiency in performing an activity of daily living (sit-to-stand transfer), and health-related quality of life. Outcome measures are assessed at baseline and at 6-, 12- and 24-months. The data collection methods for each outcome measure are described below.

#### (i) Adiposity

Weight is measured with the children barefoot and wearing light clothing, using Tanita HD646 scales (Tanita Corporation of America Inc, Illinois, USA) to 0.1 kg. Height is measured to 0.1 cm using the stretch stature method and PE87 portable stadiometers (Mentone Educational Centre, Victoria, Australia). Non-extensible steel tapes are used to assess waist circumference, which is measured at the level of the mid point between the lower costal border and the iliac crest. Anthropometric measures are conducted using the International Society for the Advancement of Kinanthropometry (ISAK) procedures [21].

#### (ii) Metabolic profile measures

Systolic and diastolic blood pressure is measured using an automated blood pressure monitor (CRITIKON, Tampa, USA) under standardised procedures. Blood is collected after the children have fasted overnight and then analysed using standard automated techniques at a single accredited pathology service (National Association of Testing Authorities, Australia, accredited). The blood will be analysed specifically for cardiovascular risk markers (cholesterol and triglycerides), glucose, insulin and C-Reactive protein.

#### (iii) Dietary intake

Two questionnaires are completed by parents concerning the child's dietary intake: a) a 137-item Food Frequency Questionnaire which has been developed for use with Australian children [22] and which is used to determine the frequency of a child's consumption of a defined list of foods over the previous 6 months; and b) the validated Child Feeding Questionnaire [20] which assesses seven factors associated with child feeding practices covering 'risk factors and concerns' and the 'control in child feeding: attitudes and practices'.

#### (iv) Fundamental movement skills and perceived competence

Fundamental movement skills proficiency is measured using the 2^nd ^edition of the Test of Gross Motor Development (TGMD-2) [23]. The children's performances of each of the 12 skills are recorded and analysed via video to allow greater measurement scrutiny. For older children (children in grade 3 and above at baseline), perceived competence is assessed using the Self-Perception Profile for Children [24], which measures six domains of perceived competence (athletic, scholastic, physical, social, behavioural, and global). For younger children (grades kindergarten, 1, and 2 at baseline) perceived competence is assessed using the pictorial version of the Self-Perception Profile, which measures four domains: cognitive and physical competence, peer and maternal acceptance [24].

#### (v) Time spent in sedentary activities

Each parent completes the Children's Leisure Activities Study (CLASS) physical activity and sedentary behaviour questionnaire on behalf of their child. This has been developed and validated for the Australian environment [25].

#### (vi) Objectively measured physical activity

Each child's physical activity is objectively measured for eight consecutive days using Manufacturing Technology Industries (MTI, MTI Health Services, Fort Walton Beach, FL, USA) 7164 accelerometers.

#### (vii) Sit-to-stand proficiency

The children's proficiency in performing an activity of daily living is characterised by the technique they use to rise from a 33 cm high chair (≈25% of standing height) from a standardised position. Riddiford-Harland et al. [26], have recently shown that lean healthy children perform this task with ease, however overweight and obese children find the task more difficult. Therefore, it is predicted that children will be able to perform activities of daily living with greater ease following the intervention. The subjects are videoed (25 Hz) in the sagittal plane, as they rise as naturally as possible for at least two trials. Two-dimensional spatial and temporal characteristics of each child's leg, thigh and trunk segment during the preparation, transition and extension phase of the rising motion are then calculated.

#### (viii) Health related quality of life

Health related quality of life is assessed using the Paediatric Quality of Life Inventory™ (PedsQL™) version 4.0. Age appropriate versions of the PedsQL™ are used for the study. Recently published US population normative data indicate high levels of internal consistency for both the self-report and parent-proxy report in this age group [27].

#### (ix) Other measures

Process data such as program adherence and implementation (discussed in detail under Quality Control), parental and participant satisfaction with each session and the whole program, and child and parent participation rates are also measured. Attendance records and adherence to home challenges are kept to determine the relationship between the number of sessions attended, time spent completing home challenges, and changes in the outcome variables.

### Recruitment

Approximately 200 families with overweight or obese children aged between 5–9 years old are being recruited, three cohorts during 2005 and one cohort during 2006 from the Hunter (population approximately 550 000) and Illawarra regions (population approximately 380 000) of New South Wales, Australia. Recruitment for each cohort commences approximately eight weeks prior to each baseline assessment. Numerous recruitment strategies are employed to enhance recruitment success and include: personalised letters sent to school principals and counsellors inviting them to promote the study within their school and in school newsletters; articles and posters placed in general practitioner (GP) surgeries, shopping centres and public libraries, 'GP Newsletters' and other GP communication networks and in pediatric dietetic departments at local hospitals; and delivery of presentations to both school principals and at pediatrician meetings. The university media units at each site distribute media releases resulting in newspaper articles and radio and television interviews and finally paid advertisements are placed in local newspapers. Interested parents are encouraged to contact the study sites directly via the study phone line. Eligibility is determined using a standardised telephone screening script, which addresses specific inclusion/exclusion criteria (Table 1). Eligible families are then sent an information sheet, detailing each intervention, their anticipated benefits, their required commitment level, the researchers involved in conducting the study and a consent form. Once written consent is obtained the participants are randomized (see Randomization section) to one of the three study arms. Participants are notified of their allocated trial group initially by telephone with confirmation by mail. Finally, recruited participants are sent a medical screen, to be completed by their GP and to be returned at the baseline assessment. Only one child from each family is able to be recruited.

**Table 1:** Inclusion and exclusion criteria for recruitment into HIKCUPS.

**Inclusion Criteria**	**Exclusion Criteria**
• Overweight/obese^1^	• Extreme obesity (BMI *Z*-score >4)
• 5.5–9 years of age	• Known syndromal cause of obesity
• Pre-pubertal (no pubic hair)	• Long term steroid use
• Generally healthy	• Medications associated with weight gain
	• Chronic illness
	• Significant dietary restrictions

^1^Defined according to age- and sex-specific international BMI cut points [29]

### Randomization

To randomly allocate participants to one of the three intervention groups the bias coin method of allocation, using a computer-based random number-producing algorithm, is used. This method ensures an equal chance of allocation to each group. Stratification by gender and site is done to ensure an equal representation in groups at each site. Only one study member at each site has access to the allocation codes and these are stored on a password-protected computer.

**Figure 1:**
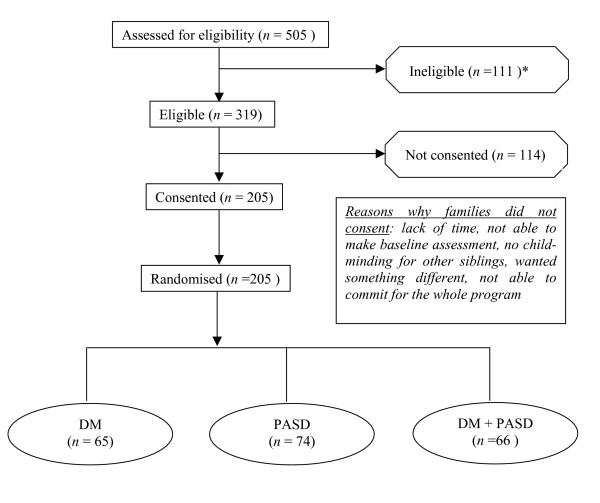
Flow of participants recruited into the HIKCUPS trial. DM = parent-centered dietary modification program, PASD = child-centered physical activity skill-development program. DM+ PASD = combined parent-centered dietary modification program and child-centered physical activity development program. (* 75 people did not wish to participate or have any further contact).

The study has no control group in the conventional sense of 'no treatment', 'waiting list' or 'typical care'. There were two compelling reasons for this: (i) every child who is overweight is deserving of an intervention and is unlikely to remain at a stable level of overweight [28] providing a strong ethical case against a non-intervention control group; and (ii) extensive recent publicity related to childhood obesity has markedly increased awareness and 'no intervention' is likely to be unacceptable to participants potentially resulting in poor retention and follow-up, thus compromising the study integrity. The flow of all participants following recruitment and randomization is summarized in Figure 1.

### Timelines for programs

The baseline, 6-, 12- and 24-month assessments and the face-to-face group sessions are delivered on the university campus at both sites. The baseline assessments, which occur approximately two weeks before the face-to-face sessions commence are conducted over two sessions. Assessments undertaken at the first session include: anthropometry; the sit-to-stand transfer; blood pressure; fasting blood collection and instruction on how to correctly wear the accelerometer. Session One is conducted early in the morning and children are offered an anaesthetic patch to minimise discomfort during blood collection and are provided with breakfast following the blood collection. Both the children and their parent's attend Session Two. The children complete the quality of life and perceived competence questionnaires and have their fundamental movement skills videoed. Parents complete four questionnaires: the Food Frequency Questionnaire; the Child Feeding Questionnaire; the sedentary behaviour and physical activity questionnaire (CLASS) and the quality of life questionnaire (PedsQL™).

**Figure 2:**
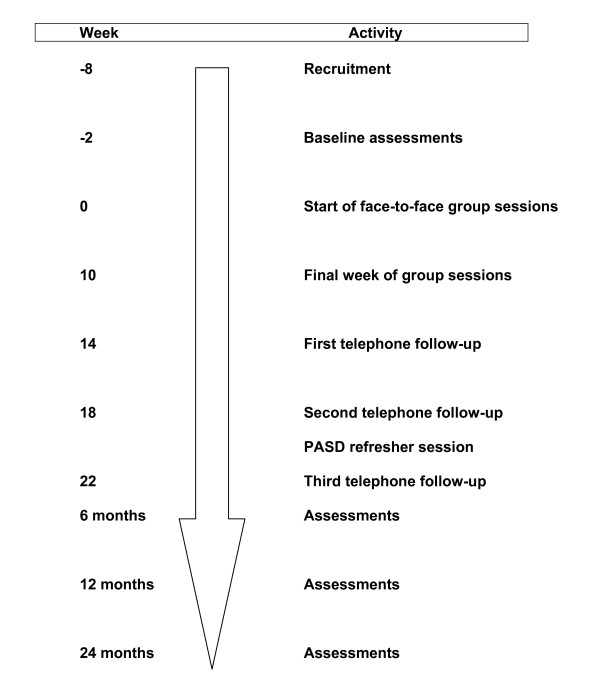
Time line for the HIKCUPS trial.

Assessments identical to those conducted at baseline are repeated at 6-, 12- and 24-months following the start of the face-to-face sessions. To maximise adherence to these sessions, participants are sent newsletters (two months before the follow-up assessments) and a parent/self-written postcard as a reminder of their program goals, and each child is also sent a birthday card. A HIKCUPS t-shirt is also given to each child at the start of the program to facilitate a sense of community with other children. Parking vouchers or permits are supplied to participants for all assessments and programs. The timeline for each cohort is shown in Figure 2.

### Sample size considerations

Our proposed sample size is based on the assumption that a 50% reduction in weight velocity over a 12-month period would be considered by the investigators as a successful outcome, changes in BMI (primary outcome variable) of 0.26–0.58 SD can be expected among children over a one-year period with the variation dependent upon age, degree of overweight, and height (Magarey, personal communication). Using the most conservative of these values (0.26 SD), a sample size of 28 participants in each of the interventions, at each site, would detect a difference in mean BMI SD score of 0.26 at baseline using an independent samples *t*-test at *p *< 0.05 with a statistical power of 0.80. Taking into consideration a 20% dropout a total of 216 participants might need to be recruited.

### Statistical analyses

Analysis will be on an intention-to-treat basis using the 6-month follow-up as the initial endpoint. Prior to analysis normality and equal variance of the data will be assessed using a Kolmogrov-Smiranov test (with Lillefors' correction) and Leven median test, respectively. Main and interaction effects for all primary and secondary outcome variables will then be determined by either multi-level modelling or a repeated measures ANOVA and simple ANCOVA with the pre-test for each group used to adjust the post-test. Co-variates that may be used are: site, sex, and child and parent program participation rates (to control for the possibility that the combined effect of DM + PASD program is not just due to an increased number of sessions with researchers). A MANOVA design will also be used to analyse the differences between groups for normal outcome variables simultaneously. Data analysis will be undertaken using SPSS (Statistical Package for the Social Sciences) with differences between treatment groups being considered statistically significant at *p *< 0.05. In addition, bivariate correlation and structural equation modelling techniques will be used to describe relationships among the various dependent and independent variables.

### Quality control

Monitoring the quality of the HIKCUPS trial is considered to be essential to ensure a robust study and to maintain internal validity. Several procedures are employed to ensure that the quality of the study is optimal, thus maximising validity and reliability of the program delivery and outcome assessments. These are described below.

#### (i) Assessor blinding

Data collection personnel are blind to participant group allocation. This is achieved by employing several external assessors not associated with the intervention and requiring them and other study full-time staff to regularly travel between study sites to test participants they have not previously met (approximately 230 km). Children and their parents are asked not to inform data collection personnel of their group allocation.

#### (ii) Written documentation

Printed and electronic copies of assessment protocols, session plans, and consent forms are stored at both sites. All written documentation, including letters sent to participants are standardised across sites subject to local institutional ethics committee approval.

#### (iii) Training

Trained physical education teachers and accredited practising dietitians run the PASD and DM programs, respectively. Data collection personnel, involved in the assessments, are trained by full-time study members prior to the assessments. Where possible the same assessors are used for all assessments.

#### (iv) Regular teleconferences

Members of the HIKCUPS study team participate in weekly teleconferences. In addition, during the first cohort, program facilitators conduct a second weekly teleconference ensuring program delivery is identical at both study sites. This additional teleconference occurs fortnightly during the second cohort. All teleconferences are minuted for reference.

#### (v) Instrument calibration

In order to ensure accurate and consistent measurements, the study scales are professionally calibrated once a year and accelerometers are calibrated every 6 months. The equipment used at each site is identical.

#### (vi) Evaluations of teaching sessions

Facilitators evaluate the coverage of the planned content of each teaching session by completing a standardized evaluation form. This ensures that the program delivery at each site is as similar as possible. The percentage of agreement (consistency) between sites in the number of activities and the percentage of adherence to scheduled number of activities is calculated.

#### (vii) Independent review

To confirm facilitator reporting of content coverage in sessions, one third of all teaching sessions, at each site, are randomly selected and reviewed by an independent reviewer. The facilitators are not informed as to when these reviews will happen. The percentage agreement between the facilitator and the independent review is calculated.

## Discussion

The study described in this paper is one of the first RCTs of its kind in Australia and important internationally. It incorporates a large sample size; has a 24-month follow-up period, thereby allowing assessment of medium-term program effectiveness; includes several important secondary outcomes (levels of physical activity and proficiency in fundamental movement skills, quality of life, diet quality and food habits) and requires only minimal, readily accessible and inexpensive equipment to assist transferability to community settings. It is a unique study that addresses many of the shortfalls in the current literature pertaining to the efficacy of childhood obesity interventions.

The results of the study will provide much needed information about the efficacy and feasibility of treatment approaches in childhood overweight and obesity. Whether or not the study ultimately yields positive long-term results, the information provided will allow other research groups to benefit from the collective experience of the study team and facilitate the implementation of well-designed RCT to address the lack of quality interventions in this important public health issue.

## Competing interests

The author(s) declare that they have no competing interests.

## Authors' contributions

RJ, TO, CC, JS, LB, PM and JW were responsible for the design of the paper and study. CC, TB, JW and JC were responsible for the development of the dietary intervention. TO, PM and DC were responsible for development of the physical activity intervention. All authors were responsible for the drafting of this paper and have read and approved the final version.

## Pre-publication history

The pre-publication history for this paper can be accessed here:


